# Comparative flow cytometry immunophenotyping of ASCs expanded in conventional flasks versus automated bioreactors

**DOI:** 10.3389/fcell.2026.1748921

**Published:** 2026-01-13

**Authors:** Hiva Alipour, Guoqiang Ren, Morten Brøndum Sørensen, Sara Aghazadeh, Zongzhe Xuan, Fereshteh Dardmeh, Simone Riis Porsborg, Trine Fink, Qiuyue Peng

**Affiliations:** 1 Regenerative Medicine Group, Department of Health Science and Technology, Aalborg University, Aalborg, Denmark; 2 The Affiliated LiHuiLi Hospital of Ningbo University, Ningbo, China

**Keywords:** adipose-derived stem cells, heterogeneity, immunophenotype, *in vitro* expansion, multichromatic flow cytometry, subpopulation selection

## Abstract

**Introduction:**

Adipose-derived stem cells (ASCs) hold significant promises for various regenerative approaches, necessitating the production of a substantial quantity of *in vitro* expanded ASCs for clinical applications. While ASC expansion is traditionally performed in tissue culture polystyrene (TCP) flasks, the Quantum Cell Expansion System, a hollow fiber bioreactor (HFB), offers an automated and closed system for cell expansion, presenting advantages over manual culture methods. In this study, we compared ASC cultures from a HFB system with traditional TCP flasks, focusing on immunophenotypes.

**Methods:**

ASCs from three donors were cultured and underwent equivalent population doublings in both systems. The cell number was counted to compare the growth rate. Furthermore, the individual expressions of 15 surface markers and their co-expression of 5 (CD73, CD90, CD105, CD166, and CD201) and 8 epitopes (CD34, CD36, CD146, CD248, CD271, CD274, and Stro-1) were analyzed by using multicolor flow cytometry.

**Results:**

ASCs expanded in the HFB and TCP system showed a comparable growth rate. Except for a significant downregulation of CD201 in HFB (*p* = 0.008), other surface marker expression profiles were largely comparable between HFB and TCP, with no statistically significant differences observed. While both systems met ISCT criteria for ASC identity, the HFB supported a broader diversity of clonal lineages (greater immunophenotypical heterogeneity), particularly by preserving both CD274-positive and -negative subpopulations. In contrast, TCP culture selectively favored CD201+ and CD274+ clones, indicating environment-driven shifts in subpopulation dynamics.

**Conclusion:**

The expansion method significantly influences the phenotypic composition of ASCs. HFB systems offer a promising alternative for large-scale ASC manufacturing by better maintaining subpopulation diversity. These findings emphasize the need for functional validation of ASC subtypes and careful consideration of expansion platforms in clinical-grade cell production.

## 1 Introduction

Recent advancements in regenerative medicine have underscored the promise of cellular therapy in restoring compromised tissues and organs (Mao and Mooney, 2015). Mesenchymal stem cells (MSCs) have emerged as promising candidates due to their versatile properties, propelling them into clinical trials for various diseases (Bateman et al., 2018). It appears that doses of several hundred million cells per patient are required to achieve optimal therapeutic effects, which require extensive vitro expansion (Kabat et al., 2020).

Traditionally, MSCs have been expanded in tissue culture polystyrene (TCP) flasks through successive trypsinization procedures upon reaching confluency (Hassan et al., 2020). As a result, most pre-clinical research on MSCs has been conducted on TCP-expanded cells, leading to well-characterized cells (Hoch and Leach, 2014). However, this method is labor-intensive and carries contamination risks, particularly during the subculturing steps (Hoch and Leach, 2014). The Quantum Cell Expansion System by TerumoBCT (United States), known as the hollow fiber bioreactor (HFB) system, has emerged as a functionally closed and automated system designed for reproducibly expanding cells in both good manufacturing practices (GMPs) and research laboratory settings (Quantum Flex, 2025). It is apparent that the HFB system has several advantages over manual cell culture, including reduced laboratory footprint with one bioreactor having a cell growth area of 2.1 m^2^ corresponding to 120 T175 TCP-flasks, significant less labor during cell culture, improved reproducibility and consistency in production as a result of better control over culture conditions such as nutrient concentrations and gas exchange, reduced risk of contamination due to the closed system, and improved scale-up potential (Quantum Flex, 2025; Barckhausen et al., 2016).

Previous studies have compared bone marrow-derived stem cells (BM-MSCs) (Jones et al., 2013) or adipose-derived stem cells (ASCs) (Haack-Sørensen et al., 2016) manufactured on the HFB system versus manual processing on TCP. These studies assessed the expression of surface markers, gene stability, expansion rate, cell yield, purity, and quality, paving the way to explore the automated HFB system as an alternative for efficient clinical-scale cell expansion (Jones et al., 2013; Haack-Sørensen et al., 2016). However, most of the studies failed to take into consideration the standardization of the population doublings (PDs) relative to the differing culture areas of the used expansion systems. This is a crucial consideration, as immunophenotypes can change along with each PD, and cells can exhibit varying growth potentials across PDs (Khong et al., 2017; Yang et al., 2018; Peng et al., 2020). Consequently, to ensure a meaningful comparison between two culture systems with a substantial difference in surface area, it is essential to perform the analysis at equivalent PDs.

Distinct immunophenotypical subpopulations have been shown to exhibit specific functional properties (Mihaila et al., 2013; Yang et al., 2013; Beckenkamp et al., 2018; Smith and Reid, 2018; Peng, 2024). To date, most of these findings are based on MSCs cultured using traditional TCP systems. In contrast, only a limited number of surface markers in ASCs cultured in HFB was reported (Haack-Sørensen et al., 2016), and the presence of immunophenotypical subpopulations in this context remains unexplored. Therefore, this comparative study of TCP and HFB culturing examined cell growth, single marker expression and highlighted the ASC subpopulations based on selected epitopes. The panels for multichromatic flow cytometry (MFC) were adapted from the previous study, including CD73, CD90, CD105, CD166, and CD201 in Panel A and CD34, CD36, D146, CD200, CD248, CD271, CD274, and Stro-1 in Panel B (Peng et al., 2022). To enable a direct comparison between the two culture systems, ASC expansion was carried out to achieve comparable PDs.

## 2 Materials and methods

### 2.1 Study design

**FIGURE 1:**
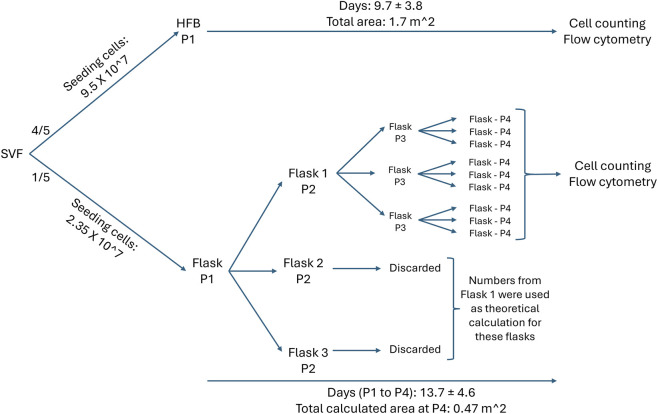
Expansion of ASCs in the flask (TCP) and bioreactor (HFB) systems. Flowchart of experimental setup. One-fifth of SVF cells harvested were used in the TCP system and the remaining four-fifths in the HFB system. The expansion was analyzed in terms of cell growth rate and surface immunophenotypical profiling. SVF, stromal vascular fraction; P, passage.

This project is an experimental study. The HFB has a considerably larger growth surface than the TCP flask (1.7 vs. 0.0175 m^2^, respectively). Therefore, it is rather challenging for ASCs to achieve the same PD from a single seeding between the two systems. Accordingly, HFB cells expanded into confluency following a single seeding with a given number of cells (Figure 1). To ensure a comparable number of PDs during both TCP and HFB expansion, four times fewer cells were seeded on a single T175 TCP flask, followed by three additional passages to reach a total of 27 T175 flasks, corresponding approximately a quarter of the bioreactor surface (0.47 m^2^). The cells in the TCP cultures were passaged at a 1:3 ratio. For practical reasons, however, two of the flasks in passage 2 (P2) were excluded (but considered in final calculations), and only one was used for further culture to reach nine flasks at P4. A flowchart of the experimental setup demonstrates the number of seeded cells and the culture period (days) in the TCP and HFB systems can be seen in Figure 1. The expansion was analyzed regarding cell growth rate and surface immunophenotypical profiling. The experiments were carried out three times independently.

### 2.2 Lipoaspirate collection and SVF isolation

This *in vitro* study included lipoaspirates (50–100 mL) collected from three healthy donors (recruited from 15 January 2020, to 29 September 2022) undergoing elective liposuction surgery (Aleris–Hamlet Private Hospital and Aalborg University Hospital, Aalborg, Denmark). The protocols have been approved by the regional committee on biomedical research ethics in Northern Jutland (project no. N-20160025), and all donors provided written informed consent prior to the surgery. Tissue collection was carried out according to the principles defined by the Declaration of Helsinki and followed the rules defined by Danish legislation on anonymized tissue (Komitélov §14).

The stromal vascular fraction (SVF) was isolated in accordance with the previous procedures (Fink et al., 2011; Peng et al., 2020). In brief, the lipoaspirates were washed and digested with 0.6 U/mL collagenase NB4 standard grade (Nordmark Biochemicals, Uetersen, Germany), followed by filtration, centrifugation, and resuspension. One-fifth of the SVF suspensions from each donor were used in the TCP system, and the remaining four-fifth was used in the HFB system (Quantum; Terumo BCT, Lakewood, Colorado, United States).

### 2.3 Cell expansion in the TCP system

The allocated SVF suspensions were added to the T175 culture flasks (Greiner Bio-one, Frickenhausen, Germany) containing alpha-Minimum Essential Medium with low glucose and GlutaMAX (Gibco, Taastrup, Denmark) supplemented with 5% heparin-free PLTGold® human platelet lysate (HPL, Sigma-Aldrich, Søborg, Denmark) and 1% antibiotic (penicillin/streptomycin; Gibco). Non-adherent cells were removed by changing the medium 2 days after seeding. The media was changed every 2–3 days until reaching 80%–90% confluency, presented as passage 1 (P1) in this study. The cells were detached by TrypLE (Gibco), and one-third of the collected cells were used for subsequent culture in one new T175 flask (P2). The cells from P2 were equally divided into three new flasks (P3). The process was repeated for each P3 flask to create nine flasks at P4. The cell yield was determined using Nucleocounter NC-200 (Chemometec, Allerod, Denmark).

### 2.4 Cell expansion in the HFB system

Prior to use, the HFB was primed with phosphate-buffered saline (PBS, Gibco) and coated with cryoprecipitate (Blood Bank, Aalborg University Hospital) as previously described by Haack-Sørensen et al. (2018). The same culture media as TCP system were used for HFB culturing.

Four-fifths of SVF suspensions were transferred into a Quantum cell inlet bag (Terumo BCT) and automatically loaded into the hollow fibers. To ensure a full attachment, cells were cultured at a relatively static condition without inlet flow, and the non-adherent cells were then washed away after 24 h. Standard ASC culture condition was an incubation temperature of 37 °C and a pre-mixed gas supply (20% 0_2_, 5% CO_2_, balanced with N_2_). ASCs were fed by a continuous supply of growth media at a rate of 0.1 mL/min. Lactate and glucose levels were measured daily using the LactatePlus Meter (Nova Biomedical, Waltham, United States) and the ContourNext Meter (Ascensia Diabetes Care, Parsippany, SA), respectively. When the lactate levels reached 3 mM, the flow rate increased to 0.2 mL/min and doubled with every 1 mM rise in lactate concentration. The harvesting program was initiated when the flow rate reached 1.6 mL/min and the lactate concentration remained above 6 mM for 24 h. To this end, the cells were dislodged with 180 mL TrypLE for 15–20 min, after which the digestion was terminated with fresh growth medium. The cell yield was determined using Nucleocounter NC-200 (Chemometech, Allerod, Denmark).

### 2.5 MFC

The calibration of MoFlo Astrios EQ cytometer (Beckman Coulter, Brea, CA, United States) and flow compensation were conducted with the aid of “Sphero ultrarainbow” six peak fluorescent particles, “Sphero ultrarainbow” single peak fluorescent particles (Spherotech, Lake Forest, IL, United States) and CompBeads plus set anti-mouse Ig, κ (BD Biosciences, Lyngby, Denmark), respectively, as described in previous publications (Nielsen et al., 2016; Peng et al., 2022).

Two panels of fluorophore-conjugated antibodies, allowing for analysis of eight and five surface markers as previously described by Peng et al. (2022), were used to maximize the number of simultaneously detectable surface markers. Cell viability and co-expression of CD73, CD90, CD105, CD166, and CD201 in Panel A and CD34, CD36, D146, CD200, CD248, CD271, CD274, and Stro-1 in Panel B, were analyzed on the trypsinized ASCs using a fixable viability dye (FVS570) and a batch of directly conjugated antibodies (all from BD Bioscience and ThermoFisher Scientific). The full panel design is shown in Table 1.

**TABLE 1:** Panel design in multicolor flow cytometry.

Laser	Channel	Fluorochrome	Panel A	Panel B
355 nm	395/25 BP	BUV395	CD201	CD36
​	525/40 BP	BUV496	​	CD34
​	740/40 BP	BUV737	​	CD248
488 nm	513/26 BP	FITC A/BB515B	CD73	CD200
​	710/45 BP	Percp-Cy5.5^a^/BB700^b^	CD90	CD271
561 nm	579/16 BP	Viability dye	FVS570	FVS570
​	614/20 BP	PE-CF594	CD105	CD274
​	785/60 BP	PE-Cy7	​	CD146
640 nm	664/22 BP	Alexa Fluor 647^a^/APC^b^	CD166	Stro-1

BP, bandpass; FVS570, fixable viability stain 570 was used in both panels.

^a^ fluorochrome conjugated antibody in Panel A.

^b^ fluorochrome conjugated antibody in Panel B.

The acquired flow data was incorporated into the Kaluza 2.1 software package (Beckman Coulter) for processing. As previously described, the basic gate strategies targeted the stable alive singlets (Peng et al., 2020; Peng et al., 2021). The positive boundary was set as the top 2.5 percent of the fluorescence minus one. Tree plots were used to identify and visualize cellular subsets.

### 2.6 Data processing and statistical analysis

Since the literature data point to ASC content range from 1% to 3% (Gentile and Sterodimas, 2020), the initial ASC seed was effectively considered 2% of the SVF input when estimating the doubling time (DT) from the first passages. The PDs were calculated using the formula “PD = 3.32 x (logXe - logXb)” (Riis et al., 2016), and the doubling time (DT) was determined using “DT = [T x (ln2)]/[ln (Xe/Xb)]”. An independent t-test was applied; when assumptions were not met, the non-parametric Wilcoxon test was used instead to compare the different outcomes between the TCP and HFB. Statistical analysis was conducted using the SPSS software (Ver. 28; IBM Corp., Armonk, NY, United States), and *p* < 0.05 was considered significant.

## 3 Results

### 3.1 Cell yield and growth rate

When looking at the TCP system, it appeared that cells grew slower in the first passage and then adapted to the *in vitro* conditions (Table 2), as evidenced by shorter DT.

**TABLE 2:** Propagation parameters of adipose-derived stem cells in the TCP system over 4 passages, calculated per T175 flask.

Passage	SVF input (per T175)	ASC yield (per T175)	PD	Days	DT	Cumulative yield*
P1	2.4 ± 1.7 × 10^7^	6.5 ± 1.1 × 10^6^	NA	6.0 ± 3.5	NA	6.5 ± 1.1 × 10^6^
P2	2.2 ± 0.4 × 10^6^	4.2 ± 1.3 × 10^6^	0.9 ± 0.2	2.3 ± 0.6	2.6 ± 0.9	1.3 ± 0.4 × 10^7^
P3	1.4 ± 0.4 × 10^6^	6.4 ± 6.2 × 10^6^	1.7 ± 1.5	2.3 ± 1.5	1.5 ± 0.6	5.7 ± 5.6 × 10^7^
P4	2.1 ± 2.1 × 10^6^	6.1 ± 2.8 × 10^6^	1.9 ± 0.8	3.0 ± 1.0	1.6 ± 0.3	1.7 ± 0.8 × 10^8^

Data are shown as mean ± SD, from three donors; SVF, stromal vascular fraction; ASC, adipose-derived stem cell; TCP, tissue culture polystyrene; PD, population doublings; DT, doubling time in days; P, passage; NA, not available. *Theoretical estimate based on total expansion from a single flask.

Cell yield and growth rates in the TCP and HFB systems have been presented in Table 2. The theoretical total cumulative cell numbers of the hypothetical complete expansion were calculated considering ASCs composed only 2% of the SVF (Gentile and Sterodimas, 2020). Although HFB showed a numerically lower DT than TCP, this difference was not statistically significant (Table 3).

**TABLE 3:** Propagation parameters (calculated per m^2^) of adipose-derived stem cells in the TCP (from passage 1–4) and HFB cultures.

ASC culture	SVF input (per m^2^)	ASC input (2% SVF input)	ASC yield (per m^2^)*	PD	Days	DT
TCP	5.0 ± 3.7 × 10^7^	1.0 ± 0.7 × 10^6^	3.5 ± 1.6 × 10^7^	8.9 ± 2.5	13.7 ± 4.6	1.52 ± 0.11
HFB	5.6 ± 4.1 × 10^7^	1.1 ± 0.8 × 10^6^	3.2 ± 2.7 × 10^7^	7.9 ± 0.6	9.7 ± 3.8	1.24 ± 0.56

Data are shown as mean ± SD, from three donors; ASC, adipose-derived stem cell; TCP, tissue culture polystyrene; HFB, hollow fiber bioreactor; SVF, stromal vascular fraction; PD, population doublings; DT, doubling time in days. *Theoretical cumulative yield on TCP, no statistical significances were observed between the two culture systems in any of the outcomes.

### 3.2 Single marker immunophenotypical patterns

**FIGURE 2:**
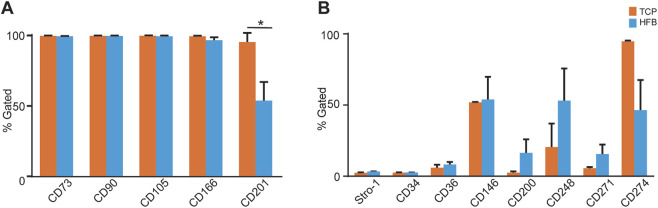
Average mean +SEM percentage of ASCs expressing single surface epitopes (in Panels **(A,B)**) from TCP and HFB cultures. TCP: tissue culture polystyrene; HFB: hollow fiber bioreactor; n = 3; *P < 0.05.

In Panel A (Figure 2A), all epitopes were highly expressed in both systems except for CD201, which was significantly downregulated in the HFB (*p* = 0.008). In Panel B (Figure 2B), three epitopes (CD200, CD248, and CD271) were higher expressed in HFB than TCP, two epitopes (CD36 and CD146) were similarly expressed in the two systems, and the single epitope CD274 was less expressed in the HFB system. The Stro-1 and CD34 were weakly expressed in both systems. Although there was a clear difference in epitope expression levels between the two culture systems in Panel B, neither reached statistical significance.

### 3.3 Complex co-expression immunophenotypical patterns

**FIGURE 3:**
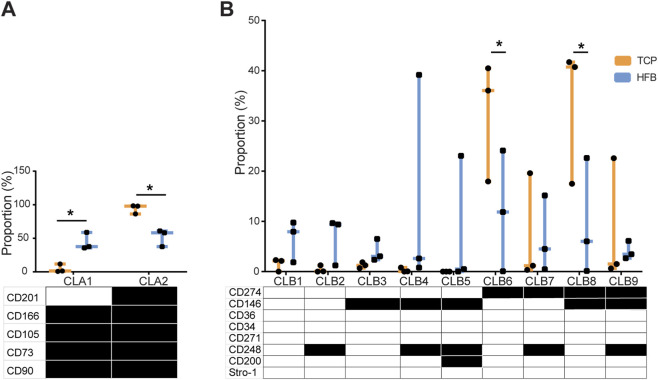
Percentage of clonal lineages in Panel **(A)** (CLA) and Panel **(B)** (CLB) of ASCs in TCP or HFB systems from three donors. Data are shown as the median ± interquartile range. Only lineages with occurrence higher than 5% in at least one donor were included. *P < 0.05. TCP: tissue culture polystyrene; HFB: hollow fiber bioreactor. A black box indicates positivity, while a white box indicates negativity.

When looking at the co-expression patterns, the five Panel A markers showed two clonal lineages (CLAs) (Figure 3A). CLA1 (CD201^–^CD166^+^CD105^+^CD73^+^CD90^+^) and CLA2 (CD201^+^CD166^+^CD105^+^CD73^+^CD90^+^) behaved reciprocally, since their system-based movement essentially mirrored the CD201 downregulation in HFB, as outlined in the previous section. As a result, the CD201 positive pattern dominated more than 90% of the TCP populations, in contrast to the HFB, where its occurrence was approximately one-half. There were no unusual patterns regarding the spread of individual values. The combinatorial patterns of the eight Panel B markers that resulted in meaningfully expressed (>5%) clonal lineages (CLBs) are shown in Figure 3B. Interestingly, HFB supported the expansion of eight to nine CLBs, while TCP culture supported only four CLB6-9. This difference could be explained by the presence of both CD274 positive and negative clones in HFB, whereas TCP culture only allowed for the expansion of CD274 positive clones. This selectivity gave rise to a statistically significant larger proportion of CLB6 and CLB8 in the TCP-expanded cells. Besides, five CLBs (CLB1-5) displayed a higher presence in HFB than the TCP, which was explained by the presence of CD274 negative cells.

## 4 Discussion

Expanding ASCs is crucial for many clinical applications, and several studies have investigated the effect of *in vitro* expansion on ASCs. Large-scale expansion using more automated systems is a practical necessity for many advanced therapy medicinal product (ATMP) productions. Nevertheless, previous studies have primarily focused on TCP systems, and the effect of HFB expansion on ASCs has been explored less. The TCP and HFB systems differ in intrinsic properties, including media replenishment, surface chemistry, topology, and the growth area as extrinsic factors. The bioreactor procedure requires only one seeding and is much simpler than the TCP protocol which requires multiple passaging. Furthermore, the culture flasks comprise a single small planar area, while the bioreactor consists of many hollow tubes, resulting in an extensive growth area.

It is well known that the immunophenotypes, homogeneity, mutagenic risk, and senescence of primary ASC cultures change with further doublings. Previous comparisons between the two systems were often established by the same passage number. However, the same passage number does not necessarily correspond to the same PD, as PD is related to the seeding density. Therefore, this study was designed to provide a comparable number of PDs during TCP and HFB expansion to allow for a more meaningful comparison. Such an approach was further validated by the result that TCP and HFB cells had a similar PD at the end of culturing. While the individual DT values on SVF are only estimates for cell growth, they still allow for a reliable comparison between the two systems. Previous studies comparing HFB- and TCP-expanded cells after two passages reported that HFB enhanced the expansion rate and yield (Lechanteur, 2014; Haack-Sørensen et al., 2016). Such accelerated cell growth by HFB system could be attributed to the continuous removal of waste materials and the reduction of abrupt fluctuations in PH and local paracrine environments facilitated by the automated feeding system. Despite difference, both systems delivered cells that met the ISCT-release criteria. However, when ensuring similar seeding density and PDs, rather than the number of passages, the present study found that growth rates of ASCs expanded in HFB and TCP were comparable. Collectively, the results indicate that initial seeding density and PD are critical regulators of growth rate.

The immunophenotypic profiles, based on surface markers, can be used as predictive markers for the biological effects of ATMPs (Lv et al., 2014). Increased immunophenotypical heterogeneity (determined by the number of clonal lineages) can lead to less predictable and more variable biological and therapeutic effects (Baer and Geiger, 2012; Prieto González, 2019). It is, therefore, crucial to investigate how these two expansion systems affect the immunophenotypes, including the individual surface markers and the clonal lineages defined by pre-selected surface markers. In this study, ASCs cultured in HFB demonstrated populations with more extensive immunophenotypical heterogeneity than TCP. When analyzing the single surface marker expression of ASCs from our 1:1 expansion comparison, both systems delivered cells that met the ISCT-release criteria for “clinical-grade” ASCs with over 95% expression of CD73, CD90, and CD105 and absent expression of hematopoietic markers like CD34 (Dominici et al., 2006), confirming results of the previous studies (Haack-Sørensen et al., 2016; Haack-Sørensen et al., 2018). Moreover, the previous study that employed both TCP and HFB cells confirmed their trilineage differentiation capacity (Ren et al., 2024).

When analyzing 13 cell surface markers individually, both systems yielded similar ASC populations, except for CD201 and CD274, that are linked to adipogenesis and immune response (Bárcia et al., 2015; Uezumi et al., 2016). When expanded in the TCP system, all cells expressed CD201 and CD274, whereas the CD201 expression was only around 50% for ASCs expanded in the HFB system. In previous studies using the TCP system, CD201 was highly expressed across eight passages of ASCs (Peng et al., 2020), suggesting that this marker may confer direct or indirect functional advantages to ASCs in TCP systems. The CD274 expression observed in this study differed from other studies, which reported expression levels below 50% for most donors (Peng et al., 2020). However, comparing these findings with other studies is somewhat challenging, as previous research has shown that surface marker expression correlates with population age (Peng et al., 2020; Peng et al., 2022). Consequently, it is crucial to consider the specific seeding and harvest designs (Dominici et al., 2006; Haack-Sørensen et al., 2016; Mastrolia et al., 2019).

Co-expression of several surface markers across the two systems showed much larger differences between the populations. For Panel A, the TCP system gave rise only to one significant clone that was positive for all five markers, whereas the HFB system gave rise to another clone that was negative for CD201. Additionally, based on Panel B, the HFB system delivered a much more heterogeneous population with a significant presence of eight individual clones, CLB1-8, compared to only four for the TCP system CLB6-9. Previous studies have demonstrated that surface marker expression in less-expanded stem cell cultures is highly heterogeneous. TCP expansion tends to select a few dominant clones, and efforts to expand specific minor clones typically result in the re-emergence of these characteristic TCP clones (Nielsen et al., 2016). Therefore, we propose that the HFB system is superior in maintaining the naive expression pattern of ASCs, preserving their *in vivo* expression to a greater extent. And, the clonal distribution was mainly driven by CD201, CD274, and CD146 expression. These altering distributions of clones are thought to support the specific biological effects induced by the donor or the culturing environment. Regarding the latter, the higher immunophenotypic diversity observed in ASCs expanded in automated HFB systems compared to TCP may result from multiple microenvironmental and mechanical factors inherent to the bioreactor design. Continuous perfusion and shear flow could provide mechanical stimuli that influence cell phenotype, while agitation or dynamic culture conditions may prevent local depletion of nutrients or accumulation of secreted factors that help to preserve subpopulation diversity. Higher initial seeding densities may enhance cell-cell interactions, and the chemical or physical properties of the hollow fiber scaffold or medium formulation may further modulate ASC behavior. Together, these factors are likely to contribute to the maintenance of a broader range of phenotypes in HFB-expanded ASCs, although the relative contributions of each remain to be experimentally determined. These hypotheses provide a framework for future studies to dissect the mechanisms underlying the observed heterogeneity.

Functional studies have previously revealed that ASCs descending from the CD274^+^CD146^+^ subpopulation were superior in terms of wound healing capacity, CD34/CD90 double-positive cells enhanced the tissue reconstruction, and CD248^+^ cells accelerated the healing process (De Francesco et al., 2009; Brett et al., 2017; Peng et al., 2022). Taking a specific example here, CD201+ cells have been reported to favor adipogenesis of mesenchymal progenitors (Uezumi et al., 2016). Therefore, it is plausible to infer that the differential proportion of HFB and TCP systems may indicate their substantial distinctions in terms of the adpogeneic potential. Collectively, this indicates distinct immunophenotypes within ASC cultures that are better suited for specific clinical scenarios than others. Of note, although phenotypic heterogeneity was identified among these two systems, functional assays were not performed to directly assess the biological properties of these subpopulations. As a result, no conclusions can be drawn regarding differences in cellular potency, differentiation capacity, immunomodulatory function, or therapeutic efficacy, etc., between those subsets harbored in the two systems. Secondly, the observed phenotypic differences were evaluated at the population level, and their stability over prolonged expansion or following *in vivo* transplantation was not examined. Finally, while prior studies have suggested potential functional relevance of these markers, the present findings are descriptive and should be interpreted as hypothesis-generating, which warrant further investigation in future.

In a pervious study (Ren et al., 2024), we reported comparable effects of ASC harvested from these two expansion systems on tri-lineage differentiation, colony-forming capacity, proliferation rate, as well as fibroblasts migration and proliferation. Building on these findings, future studies incorporating more potency testing will be required to establish whether the observed phenotypic differences translate into meaningful biological or clinical effect.

## 5 Conclusion

This study provides valuable insights into the impact of bioprocessing on ASCs during expansion in HFBs compared to TCP flasks. Our findings revealed that HFB-expanded ASCs demonstrated superior immunophenotypical heterogeneity. Regarding the impact of the culture system on the selection of specific lineages, the CD201^+^ and CD274^+^ clones were favored in the TCP conditions. In contrast, the HFB environment supported both CD274 positive and negative populations. Nevertheless, both systems met ISCT criteria for the single surface marker profiles of ASCs. These results contribute to the growing body of knowledge on large-scale cell expansion methods, specifically in regenerative medicine and cellular therapy. The study highlights the need for further exploration of the HFB system for efficient clinical-grade cell expansion, emphasizing the need for potency assays and a deeper understanding of the link between surface markers and the functionalities of ASCs in the context of ATMP productions.

## Data Availability

The original contributions presented in the study are included in the article/supplementary material, further inquiries can be directed to the corresponding author.
